# Taylor on Sexual Disorders

**Published:** 1905-09

**Authors:** 


### *BOOK REVIEWS.*

TAYLOR ON SEXUAL DISORDERS. A Practical Treatise on Sexual Disorders im Male and Female. By Robert W. Taylor, A.M., M.D., Clinical Professor of Genito-Urinary and Venereal Diseases in the College of Physicians and Surgeons (Columbia University), New York. New (3d) edition, enlarged and thoroughly revised. In one octavo volume of 575 pages with 130 engravings and 16 colored plates. Cloth, $3.00, net. Lea Brothers & Co., Philadelphia and New York, 1905.

It is with pleasure that we review the third edition of this valuable work. Dr. Taylor is a man of much force and ability and has done more than any other man in placing sexual disorders upon a rational basis free from the time-worn "unscientific conglomeration of symptoms" that fill many of the older books upon this subject.

The thorough discussion of the anatomy and physiology of the sexual organs prepares the reader in a logical way to comprehend the symptoms and pathology, and in this manner to locate accurately the disease so rationa l curative measures may be instituted. No man deserves more credit in assisting in removing the much-dreaded and little understood "bugaboo," "loss of semen," nocturnal pollution, etc., than Dr. Taylor, who has sought the actual pathology in each case and has treated the disease, and not symptoms. He has properly lain great stress upon chronic inflammations of the deep urethra and prostate, as causative factors in many of the conditions considered neurotic or of vague indefinite origin or probably due to early self-abuse, etc.

If careful examinations were made and his directions for carrying out local treatment were applied to these cases instead of ruining their digestive apparatus by nausaeting drugs there would be a vast decrease in the number of sexual neurasthenics.

We commend the above work as one of the best upon this subject in the English language.

The change in the spelling of the word "Yohimbin" to "Zohimbin" may be a typographical error, otherwise we are not aware of the reason for this spelling.

